# Efficacy and safety of atypical antipsychotic drugs (quetiapine, risperidone, aripiprazole and paliperidone) compared with placebo or typical antipsychotic drugs for treating refractory schizophrenia: overview of systematic reviews

**DOI:** 10.1590/S1516-31802010000300007

**Published:** 2010-05-06

**Authors:** Tamara Melnik, Bernardo Garcia Soares, Maria Eduarda dos Santos Puga, Álvaro Nagib Atallah

**Affiliations:** I PhD. Psychologist, researcher and professor of Internal Medicine and Evidence-Based Medicine at the Universidade Federal de São Paulo (Unifesp) and Brazilian Cochrane Center, São Paulo, Brazil.; II MD. Psychiatrist and researcher at the Brazilian Cochrane Center, Universidade Federal de São Paulo (Unifesp), São Paulo, Brazil.; III Research assistant at the Brazilian Cochrane Center, and doctoral student in the Discipline of Emergency Medicine and Evidence-Based Medicine, Universidade Federal de São Paulo — Escola Paulista de Medicina (Unifesp-EPM), São Paulo, Brazil.; IV MD, MSc, PhD. Nephrologist. Director of the Brazilian Cochrane Center, and titular professor of Evidence-Based Medicine and Emergency Medicine, Universidade Federal de São Paulo — Escola Paulista de Medicina (Unifesp-EPM), São Paulo, Brazil.

**Keywords:** Schizophrenia, Antipsychotic agents, Dopamine antagonists, Aripiprazole [substance name], Quetiapine [substance name], Risperidone, Esquizofrenia, Agentes antipsicóticos, Antagonistas de dopamina, Risperidona, Revisão

## Abstract

**CONTEXT AND OBJECTIVE:**

According to some cohort studies, the prevalence of refractory schizophrenia (RS) is 20-40%. Our aim was to evaluate the effectiveness and safety of aripiprazole, paliperidone, quetiapine and risperidone for treating RS.

**METHODS:**

This was a critical appraisal of Cochrane reviews published in the Cochrane Library, supplemented with reference to more recent randomized controlled trials (RCTs) on RS. The following databases were searched: Medical Literature Analysis and Retrieval System Online (Medline) (1966-2009), Controlled Trials of the Cochrane Collaboration (2009, Issue 2), Embase (Excerpta Medica) (1980-2009), Literatura Latino-Americana e do Caribe em Ciências da Saúde (Lilacs) (1982-2009). There was no language restriction. Randomized controlled trials, systematic reviews and meta-analyses evaluating atypical antipsychotics for treating RS were included.

**RESULTS:**

Seven Cochrane systematic reviews and 10 additional RCTs were included in this review. The data generally showed minor differences between the atypical antipsychotics evaluated and typical antipsychotics, regarding improvement in disease symptoms, despite better adherence to treatment with atypical antipsychotics. Risperidone was specifically evaluated in patients with RS in one of the systematic reviews included, with favorable outcomes, but without definitive superiority compared with other drugs of proven efficacy, like amisulpride, clozapine and olanzapine.

**CONCLUSIONS:**

The findings underscore the difficulty in treating these patients, with high dropout rates and treatment patterns of modest improvement in assessments of effectiveness. Atypical antipsychotics have advantages over typical antipsychotics mainly through their better safety profile, which leads to better adherence to treatment. A combination of antipsychotics may also be an option for some refractory patients.

## RESUMO

**CONTEXTO E OBJETIVO:**

De acordo com alguns estudos de coorte, a prevalência da esquizofrenia refratária (ER) está entre 20-40%. Nosso objetivo foi avaliar a efetividade e segurança de aripiprazol, paliperidona, quetiapina e risperidona no tratamento da esquizofrenia refratária.

**MÉTODOS:**

Avaliação crítica das revisões Cochrane publicadas na Biblioteca Cochrane e complementação com referências de ensaios clínicos randomizados (ECRs) mais atualizados sobre ER. As seguintes bases de dados foram pesquisadas: Medline (Medical Literature Analysis and Retrieval System Online) (1966-2009), Ensaios Controlados da Colaboração Cochrane (2009, edição 2), Embase (Excerpta Database) (1980-2009), Lilacs (Literatura Latino-Americana e do Caribe em Ciências da Saúde) (1982-2009). Não houve restrição a idiomas. Ensaios clínicos randomizados, revisões sistemáticas e metanálises que avaliaram antipsicóticos atípicos no tratamento da esquizofrenia refratária foram incluídos.

**RESULTADOS:**

Sete revisões sistemáticas Cochrane e 10 ECRs complementares foram incluídos nessa revisão. No geral os dados demonstram pequenas diferenças entre os antipsicóticos atípicos avaliados e os típicos na melhora dos sintomas da doença, apesar da melhor adesão ao tratamento com os atípicos. A risperidona foi avaliada especificamente em pacientes com esquizofrenia refratária em uma das revisões sistemáticas incluídas, a qual demonstrou desfechos favoráveis, porém não definitivos quando comparada a drogas também com eficácia comprovada como amisulprida, clozapina e olanzapina.

**CONCLUSÕES:**

Os dados reforçam a dificuldade de tratar esses pacientes, com elevadas taxas de desistência do tratamento e padrões de melhora modestos nas avaliações de eficácia. Os antipsicóticos atípicos têm vantagens sobre os típicos principalmente pelo melhor perfil de segurança, o que leva a melhor adesão ao tratamento. A associação de antipsicóticos também pode ser uma opção em alguns pacientes refratários ao tratamento.

## INTRODUCTION

### Refractory schizophrenia

Schizophrenia affects about 1% of the population and accounts for 25% of psychiatric hospitalizations. Its course is variable, with about 30% of cases presenting almost complete recovery; 30% in remission with incomplete and partial loss of function; and 30% with significant and persistent deterioration of professional, social, and emotional functionality.

Approximately one-third of patients with schizophrenia do not respond to first-generation antipsychotics.^1^ According to some cohort studies, the prevalence of refractory schizophrenia (RS) is between 20 and 40% of schizophrenia patients.^2^ Some authors have reported higher rates; however, it has been questioned whether these findings might represent inappropriate treatment strategies regarding dose or duration of treatment.

It remains controversial in the literature regarding whether RS can be considered to be a more chronic and more severe subtype of schizophrenia, characterized by refractory positive symptoms and poor social integration, or whether it must be understood as a distinct subtype of schizophrenia with well-defined neurochemical and anatomical characteristics. Using magnetic resonance imaging, dopaminergic, serotonergic and glutamatergic changes in the brain have been documented in patients with RS, compared with patients who responded well to treatment.^3^

Some clinical features appear to be associated with RS. Patients with RS present greater severity of psychopathology (delusions and hallucinations), have higher hospital admission recurrence rates, use more mental health resources, have poorer quality of life and pose a greater financial burden, both because they are removed from productive life and because they need help from others who, in turn, also see their work capacity reduced. Meltzer et al.,^4^ comparing patients with RS and non-RS, reported that patients with RS had an age at onset that was two years younger, and were more often men. Similarly, Henna et al.^5^ observed that in terms of distribution between the genders, RS patients were predominantly male, had more hospitalizations and an onset at around 17 years of age, whereas those with non-refractory schizophrenia at an onset at around 20 years of age. Because RS patients are refractory to ordinary doses of typical antipsychotics, these patients are usually treated with doses that are much higher than the usual doses of medication, and with polypharmacy.

### Conceptual aspects of RS

There is a consensus in the literature regarding the core feature of RS: persistence of moderate to severe positive symptoms. Barnes et al.^2^ reported that other dimensions of schizophrenia should be taken into account in diagnosing RS, such as negative symptoms and cognitive attributes, as well as the inability to return to a higher level of premorbid functioning.

In 2002, the National Institute of Clinical Excellence^6^ defined RS, highlighting the characteristic of absence of satisfactory clinical improvement despite sequential use of recommended doses of at least two antipsychotics for 6-8 weeks (and one of the drugs had to belong to the second generation of antipsychotics), with assessments carried out using scales such as the Brief Psychiatric Rating Scale (BPRS) and the Clinical Global Impression (CGI). Clinically, the multidimensional notion of RS may be useful, since it acknowledges the presence of persistent functional disability and psychosocial functioning disability despite adequate treatment, thus causing inadequate definition in terms of a simple dichotomous (yes or no) determination.

Some authors have tried to construct one-dimensional definitions based on overall reduction of symptoms or two-dimensional definitions, taking into account the social adaptation and the reduction of symptoms. Barnes et al.^2^ and Brenner et al.^7^ defined RS as continuous resistance to treatment, and developed a scale based on psychopathology and social adaptation. The operational criterion most widely used for defining RS in clinical studies is that of Kane et al,^8^ which was used in the study that introduced the therapeutic formulation clozapine into schizophrenia. The criterion of Kane et al.^8^ is three-dimensional: 1) History: a history of partial or total lack of response to previous treatment using two antipsychotics at adequate doses and for adequate periods; 2) Current (severity of symptoms): the patient must present a severe degree of psychopathology, as assessed by the BPRS and the CGI; and 3) Confirmatory: after treatment with one or more antipsychotic medications, the patient must show minimal improvement in symptoms (BPRS and CGI), compared with the levels of psychopathological conditions prior to treatment.

## OBJECTIVES

The objective of this study was to undertake a systematic review to evaluate the effectiveness and safety of atypical antipsychotics (aripiprazole, paliperidone, quetiapine and risperidone), compared with other antipsychotics or associations of interventions for treating refractory schizophrenia.

## REVIEW METHODS

### Criteria for study inclusion

#### Types of studies

Randomized controlled trials (RCTs) and systematic reviews (SRs) of randomized controlled trials were included. Quasi-randomized studies, defined as studies using inadequate allocation, such as date of birth, day of week, or assignment of subjects to alternative treatments were included in the sensitivity analysis in order to assess effectiveness.

Studies published as abstracts were included when sufficient information on methods and results were provided. For questions or incomplete data, the main authors were contacted and interviewed to obtain additional information or complement the existing data.

#### Types of participants

Patients with refractory schizophrenia were included. Randomized clinical trials that evaluated patients with refractory and non-refractory schizophrenia and did not distinguish between these groups in the analysis were not included.

#### Types of interventions

- Intervention: aripiprazole, paliperidone, quetiapine or risperidone, alone or combined with other interventions.
- Control group: typical antipsychotics, atypical antipsychotics, placebo, different doses of antipsychotics, discontinuation or intermittent treatment.

#### Types of outcomes

Primary outcomes:

- Improved overall psychopathology: scales such as PANSS (Positive and Negative Syndrome Scale), BPRS and CGI;
- Adherence to treatment: time until discontinuation of medication, frequency of treatment discontinuation and reasons for discontinuation.
- Improvement of specific symptoms: positive, negative or cognitive.

Secondary outcomes:

- Suicide.
- Specific mortality, i.e. mortality due to schizophrenia.
- Relapse: appearance of positive and negative symptoms.
- Need for hospital admission.
- Incidence of adverse events.
- Quality of life.
- Cost analysis.

### Search strategy for studies

Systematic reviews of the medications evaluated in this review were identified in the Cochrane Library. Since these reviews were not up to date, we conducted an additional search for randomized clinical trials that met the inclusion criteria. There was no language restriction, and studies were included whether published or not.

The date of the search was specific for each product, taking into consideration the date of the search in the systematic review assessed:

- Aripiprazole: November 2007^9,10^
- Paliperidone: December 2006^11^
- Quetiapine: February 2003^12^
- Risperidone: January 2002^13-15^

#### - Electronic databases

In association with a specific search phrase for randomized clinical trials, validated for each database, we used the following search strategy for this review:

(schizophrenia * AND (OR resistant OR refractory non-response *)) AND ((* OR abifity aripiprazole) OR (quetiapine seroquel OR *) OR (risperidone consult * OR) OR (paliperidone INVEGA * OR)).

The search strategy for clinical trials in Medline, via the PubMed interface (1966-2009) was the following:

(randomized controlled trial [pt] OR controlled clinical trial [pt] OR randomized controlled trials [mh] OR random allocation [mh] OR double-blind method [mh] OR single-blind method [mh] OR clinical trial [pt] OR clinical trials [mh] OR ("clinical trial" [tw]) OR ((singl * [tw] OR double * [tw] OR Trebl * [tw] OR tripl * [tw]) AND (mask * [tw] OR blind * [tw])) OR (placebos [mh] OR placebo * [tw] OR random * [tw] OR research design [mh: noexp] OR comparative study [mh] OR evaluation studies [mh] OR follow-up studies [mh] OR prospective studies [mh] OR control * [tw] OR prospectiv * [tw] OR volunteer * [tw]) NOT (animals [mh] NOT human [mh])).

The search strategy for clinical trials in Embase via Ovid (1980-2009) was the following:

Randomized controlled trial/ 2. Controlled study/ 3. Randomization/ 4. Double blind procedure/ 5. Single blind procedure/ 6. Clinical trial/ 7. (clinical trial $ adj5). ti, ab, hw. 8. ((doubl $ or singl $ or tripl $ or $ Trebl) adj5 (blind $ or mask $)). ti, ab, hw. 9. Placebo / 10. Placebo $. Ti, ab, hw. 11. Random $. Ti, ab, hw. 12. Methodology.sh. 13. latin square.ti, ab, hw. 14. crossover.ti, ab, hw. 15. cross-over.ti, ab, hw. 16. Crossover Procedure/ 17. Drug comparison/ 18. Comparative study/ 19. (comparative adj5 trial $). ti, ab, hw. 20. (control $ or prospectiv $ or volunteer $). ti, ab, hw. 21. exp "Evaluation and Follow Up"/ 22. Prospective study/ 23. or/1-22 24. animal/no (human/and animal/) 25. 23 not 24.

The search strategy for clinical trials in the Lilacs (Literatura Latino-Americana e do Caribe em Ciências da Saúde) (1982-2009) was the following:

((Pt randomized controlled trial OR Pt controlled clinical trial OR Mh randomized controlled trials OR Mh random allocation OR Mh double-blind method OR Mh single-blind method) AND NOT (Ct animal AND NOT (Ct human and Ct animal)).

#### - Websites visited

- http://www.controlledtrials.com
- http://clinicaltrials.gov/ct/gui
- http://www.CenterWatch.com/
- http://www.fda.gov

#### - List of references

References relating to trials and review articles that were identified were evaluated, in order to locate any additional studies not found in the databases.

### Study selection

Two reviewers independently assessed the titles and abstracts of all articles identified in the search, and evaluated the full texts of articles that described studies that might potentially be included in this review.

### Assessment of methodological quality

The methodological quality, defined as the degree of confidence that the design and reporting of the study were free from bias,^16^ was evaluated by the authors, taking into account the results from the Cochrane systematic reviews included. For additional randomized clinical trials that were analyzed, the randomization process was the main methodological criterion evaluated.

### Data analysis

The main meta-analyses in the systematic reviews were discussed and complemented with individual studies that evaluated patients with refractory schizophrenia. All the tests were performed using the intention to treat method, and all randomized patients were included in the analysis, regardless of their adherence or outcome.

## ARIPIPRAZOLE FOR TREATING REFRACTORY SCHIZOPHRENIA

Aripiprazole is an atypical antipsychotic for which the mechanism of action differs from other atypical antipsychotics (e.g. clozapine, olanzapine, quetiapine, risperidone and ziprasidone). Aripiprazole appears to exert its effects, in principle, on the D2 receptor partial agonist, which modulates dopaminergic activity in areas where dopamine activity may be increased or decreased, like the mesolimbic brain area.^9^ In addition to its partial agonist action on D2 receptors, aripiprazole is also a partial agonist of the 5-HT1A receptor.^9^

Two Cochrane systematic reviews have evaluated the use of aripiprazole for treating schizophrenia.

### Cochrane review of aripiprazole for treating schizophrenia

The review by El-Sayeh and Morganti^9^ was a comprehensive review that aimed to evaluate the effects of aripiprazole in treating general schizophrenia, compared with no intervention control. Fifteen studies were included, which evaluated 7,110 individuals.

**Figure 1.**
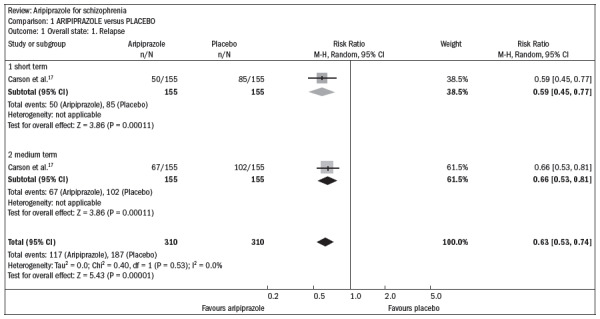
Efficacy of aripiprazole versus placebo, in relation to the outcomes of overall state and relapse, over the short and medium terms.

Compared with the placebo, aripiprazole significantly reduced the relapse rate over the short and midterm (n = 310; one RCT; relative risk, RR 0.66; confidence interval, CI 0.5 to 0.8; number needed to treat, NNT 5; CI 4-8) (Figure 1).^17^

Aripiprazole promoted greater adherence to treatment, compared with the placebo (n = 2271; 8 RCTs; RR 0.72; CI 0.5 to 0.97; NNT 26; CI 16-239).

However, no differences were found in relation to the overall state and the rate of dropout, in comparing aripiprazole with risperidone and olanzapine.

The rates of adverse events were also similar, with the exception of less elevated prolactin (n = 301, one RCT, RR 0.04 CI 0.02 to 0.1, NNT 2 CI 1 to 2.5) and lower QT prolongation medium (30 mg/day) (n = 200; one RCT; weighted mean difference, WMD -10.0; CI -16.99 to -3.0), comparing aripiprazole with risperidone.

**Figure 2.**
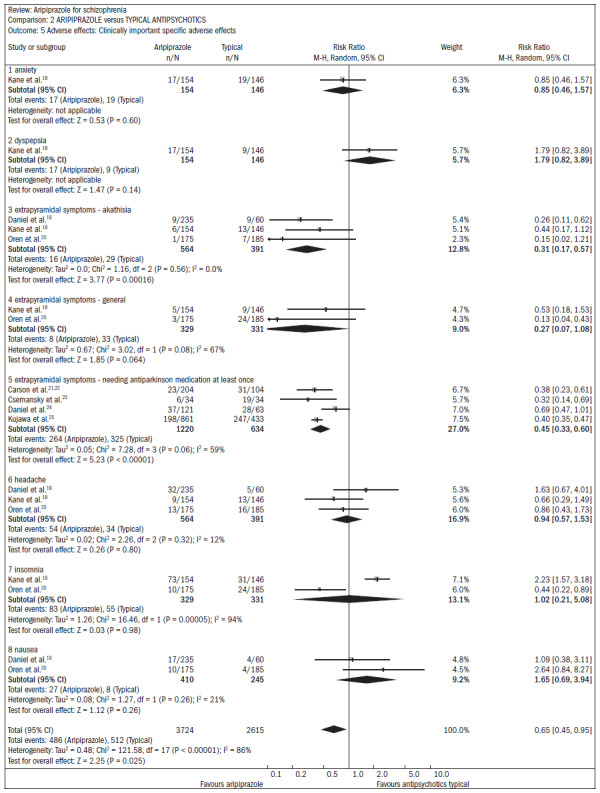
Aripiprazole versus typical antipsychotics, in relation to adverse effects.

Compared with typical antipsychotics, there were no benefits for aripiprazole in relation to the overall state, mental state, or quality of life. Both groups had similar rates of adverse events, with the exception of akathisia (n = 955; RR 0.31; CI 0.2 to 0.6; NNT 20; CI 17 to 32) and the need for antiparkinsonian medication (n = 1854; four RCTs; RR 0.45; CI 0.3 to 0.6; NNT 4; CI 3 to 5). These effects were less frequent in patients taking aripiprazole (Figure 2).^18-25^

The results from five new studies identified through updating this review did not significantly alter the main results and the initial findings of this review.

## CONCLUSION

Aripiprazole is effective for treating schizophrenia, but not so different from typical and atypical antipsychotics with regard to treatment response, efficacy or tolerability. Compared with typical antipsychotics, aripiprazole presents a lower risk of akathisia, and compared with atypical antipsychotics, lower risk of increased prolactin levels and QTc interval prolongation.

### Cochrane review of aripiprazole compared with typical antipsychotics for treating schizophrenia

A second Cochrane review^10^ that specifically evaluated aripiprazole in comparison with typical antipsychotics for treating schizophrenia was updated. Nine randomized trials involving 3,122 individuals that compared aripiprazole with typical antipsychotics were included. None of the studies reported the relapse rate. The dropout rate was high, as were the numbers of records with incomplete data.

**Figure 3.**
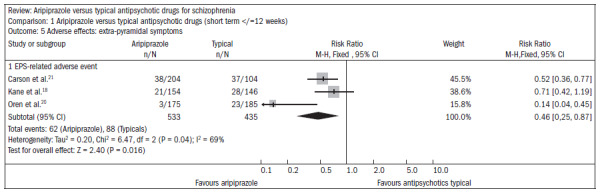
Aripiprazole versus typical antipsychotics, in relation to extrapyramidal symptoms

Patients using aripiprazole showed extrapyramidal symptoms less frequently than did those using typical antipsychotics (n = 968; three RCTs; RR 0.46; CI 0.3 to 0.9; NNT 13; CI 17 to 10) or, in particular, akathisia (n = 897; three RCTs, RR 0.39; CI 0.3 to 0.6; NNT 11; CI 9 to 14) (Figure 3).^18,20,21^

The occurrence of hyperprolactinemia was lower in patients taking aripiprazole, compared with those taking typical antipsychotics (n = 300; one RCT; RR 0.07; CI 0.03 to 0.2; NNT 2; CI 3 to 1). Patients taking aripiprazole had lower risks of sinus tachycardia (n = 289; one RCT; RR 0.09; CI 0.01 to 0.8; NNT 22; CI 63 to 13) and blurred vision (n = 308; one RCT; RR 0.19; CI 0.1 to 0.7; NNT 14; CI 25 to 10), but increased risks of dizziness (n = 957. three RCTs; RR 1.88; CI 1.1 to 3.2; number needed to harm, NNH 20; CI 33 to 14) and nausea (n = 957; three RCTs; RR 3.03; CI 1.5 to 6.1; NNH 17; CI 25 to 13. The dropout rates were high in both groups, although significantly more participants in the aripiprazole group completed the study, over the long term (n = 1294; one RCT; RR 0.81; CI 0.8 to 0.9; NNT 8; CI 5 to 14).

### Review conclusion

Aripiprazole differs little from typical antipsychotics with regard to efficacy. However, it has significant advantages in terms of tolerability. Further studies are needed in order to replicate and validate these results and determine the effectiveness of aripiprazole in clinical practice.

### Studies of refractory schizophrenia

From the updated search, two RCTs were selected that were not included in the systematic reviews mentioned above, which evaluated the effectiveness of aripiprazole for treating refractory schizophrenia.

Chang et al.^26^ conducted a randomized, double-blind study evaluating the efficacy and safety of aripiprazole associated with clozapine among patients with refractory schizophrenia. Patients diagnosed with schizophrenia according to the DSM-IV (Diagnostic and Statistical Manual of Mental Disorders) and with a history of treatment failure or partial response to long-term use of clozapine were recruited. Sixty-two patients evaluated using the Brief Psychiatric Rating Scale (BPRS) (scoring at least 35) and SANS (Scale for the Assessment of Negative Symptoms) were randomly assigned to one of two groups: aripiprazole associated with clozapine (5-30 mg/day) or placebo for eight weeks. The primary outcome assessed was a change in the total BPRS score at baseline and no significant difference in this outcome between the two groups. In secondary analysis, the improvement was significantly greater with aripiprazole associated with clozapine than with a placebo, in relation to negative symptoms assessed using the negative symptom subscales of the BPRS and SANS scales (total score). Prolactin levels and triglycerides were significantly lower in the aripiprazole group than in the placebo group. There was no significant difference between the two groups in relation to adverse effects, including extrapyramidal symptoms and hyperglycemia. The authors concluded that although aripiprazole associated with clozapine did not lead to significant improvement in all symptoms of schizophrenia, a favorable change in areas relating to negative symptoms was observed.

Kane et al.^27^ conducted a randomized, multicenter, double-blind trial comparing the efficacy and safety of aripiprazole and perphenazine in patients with refractory schizophrenia. They included patients diagnosed according to the DSM-IV and with a history of resistance to treatment. Patients received four to six weeks of open treatment with olanzapine or risperidone to confirm treatment resistance. Only patients who completed this open period without response (< 20% improvement in subscales relating to the positive and negative syndrome scale (PANSS) total score or Clinical Global Impression Score ≥ 4) were included in the six weeks of double-blind treatment. In all, 300 patients with confirmed resistance to treatment were randomized into two groups: aripiprazole (15-30 mg/day) or perphenazine (8-64 mg/day). The primary outcome was measured as changes in the PANSS scores. Both groups (aripiprazole and perphenazine) showed significant clinical improvement according to PANSS total scores. After six weeks, 27% of the patients treated with aripiprazole and 25% of the patients treated with perphenazine responded to treatment (≥ 30% reduction in PANSS total score or a Clinical Global score of 1 or 2). Patients treated with perphenazine reported higher incidence of extrapyramidal symptoms and higher prolactin levels than in patients treated with aripiprazole (57.7% versus 4.4%, P < 0.001). The improvement in quality of life was considered clinically relevant according to the results (≥ 20% improvement in score on the quality-of-life scale). This was found in 36% of patients treated with aripiprazole and 21% of those treated with perphenazine (P = 0.052). The authors concluded that at the doses described in this study, aripiprazole and perphenazine may improve the symptoms of refractory schizophrenia in patients who have not responded to olanzapine or risperidone.

## PALIPERIDONE FOR TREATING REFRACTORY SCHIZOPHRENIA

Paliperidone is the active metabolite of risperidone, and is used as an atypical antipsychotic oral medication or as an injectable depot for monthly usage.

### Cochrane Review on paliperidone for treating schizophrenia

In the review by Nussbaum and Stroup,^11^ five RCTs comparing oral paliperidone with placebo (n = 1647) and three RCTs comparing with olanzapine, quetiapine, risperidone (n = 1332) were included. No RCTs studying injections of paliperidone with prolonged action were identified.

**Figure 4.**
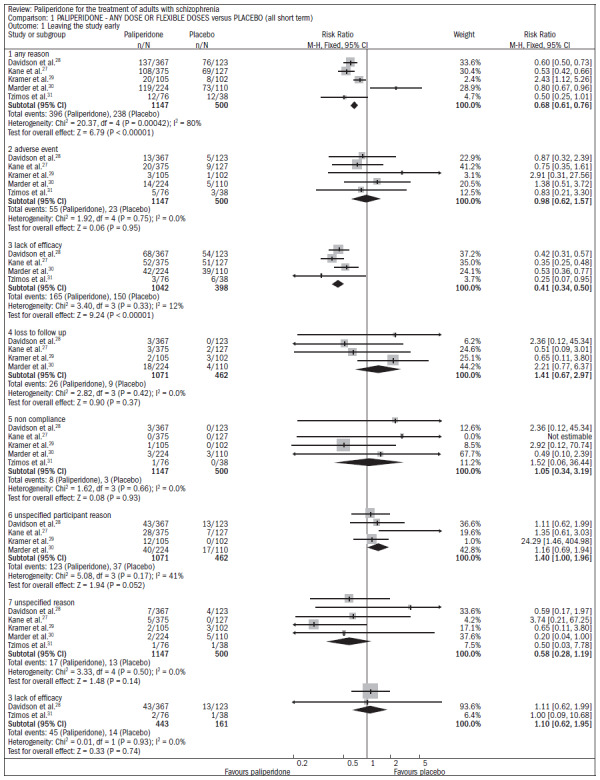
Paliperidone versus placebo, in relation to dropout rate in the study.

Compared with placebo, the patients receiving paliperidone had a lower dropout rate in the study (n = 1647; five RCTs; RR 0.68; CI 0.61 to 0.76; NNT 7; CI 6 to 9) (Figure 4).^27-31^

**Figure 5.**
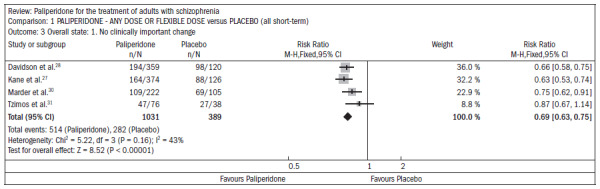
Efficacy of paliperidone versus placebo, in relation to improvements in general condition and disease symptoms.

The patients taking paliperidone had higher rates of improvement in general condition and disease symptoms, compared with placebo (n = 1420; four RCTs; RR 0.69; CI 0.63 to 0.75; NNT 5; CI 4 to 6) (Figure 5).^27,28,30-31^

**Figure 6.**
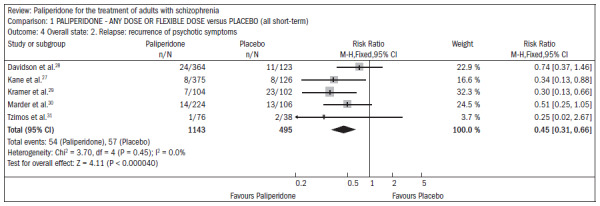
Efficacy of paliperidone versus placebo, in relation to recurrence of psychosis.

The use of paliperidone resulted in lower recurrence of psychosis (n = 1638; five RCTs; RR 0.45; CI 0.31 to 0.66; NNT 16; CI 13 to 26) (Figure 6).^27-31^

With regard to adverse events, in comparison with placebo, paliperidone resulted in:

- higher incidence of tachycardia (n = 1638; five RCTs; RR 1.88; CI 1.3 to 2.86; NNH 21; CI 11 to 90);
- significantly higher elevation of prolactin, for both men (n = 413, 4 RCTs, WMD 27.68; CI 23.66 to 31.69) and women (n = 252, 4 RCTs, WMD 87.39; CI 74.27 to 100.51);
- higher rate of extrapyramidal disorders (n = 1638; five RCTs; RR 2.21; CI 1.3 to 3.9; NNH 28; CI 12 to 129);
- greater weight gain (n = 769, 4 RCTs, WMD 1.07; CI 0.65 to 1.49; I^2^ index 78%) in the meta-analysis of three RCTs comparing paliperidone with olanzapine. The loss rate during the studies was similar in both groups of patients (around 40% over six weeks of study).

**Figure 7.**
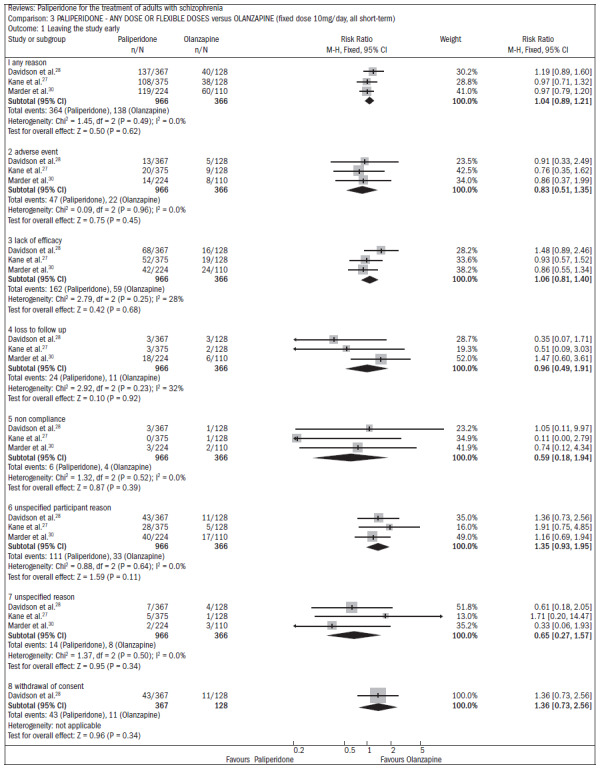
Efficacy of paliperidone versus olanzapine, in relation to improvement of symptoms over a six-week period.

In assessing the improvement of symptoms over the six-week period, there was no significant difference favoring olanzapine in the PANSS assessment (n = 715; DMP 2.44; CI -0.52 to 5.35) (Figure 7).^27,28,30^

Regarding the profile of adverse events, the main differences between the use of paliperidone and olanzapine occurred in relation to weight gain and movement disorders. The group taking paliperidone had lower weight gain (n = 660; three RCTs; DMP -0.88; CI -1.38 to -0.37).

**Figure 8.**
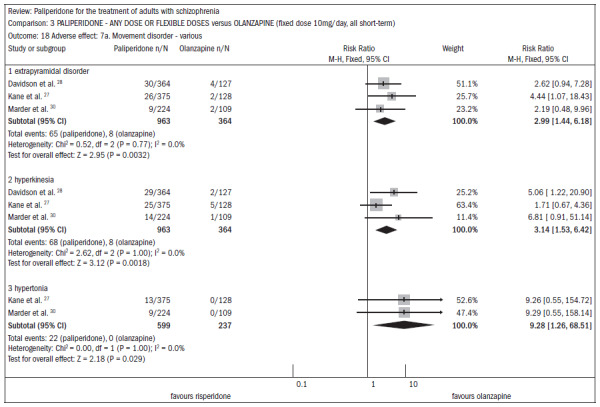
Paliperidone versus olanzapine, in relation to adverse effects.

Patients taking olanzapine had a lower rate of movement disorders (Figure 8):^27,28,30^

- extrapyramidal disorders (n = 1327; three RCTs; RR 2.99; CI 1.4 to 6.2);
- hyperkinesis (n = 1327; three RCTs; RR 3.14; CI 1.5 to 6.4);
- stiffness (n = 1327; two RCTs; RR 9.28; CI 1.3 to 68.5).

### Conclusions from review

In short-term studies, oral paliperidone is more effective than placebo. Its safety profile is similar to that of risperidone, with movement disorders, weight gain and tachycardia more common with paliperidone than with placebo. In addition, paliperidone is associated with significant increases in serum prolactin, which can cause sexual dysfunction, although this outcome was not assessed in this review because of limited data from the individual studies. At a dose of 3 mg/day, oral paliperidone appears to have efficacy similar to oral olanzapine 10 mg/day. There is a need for studies comparing paliperidone with risperidone because of the great similarity of these drugs.

### Studies on refractory schizophrenia

The search for RCTs did not identify any studies on paliperidone used for patients with refractory schizophrenia. However, two RCTs other than those included in the Cochrane review are presented below. Turkoz et al.^32^ evaluated the effect of paliperidone on negative symptoms of schizophrenia, through a meta-analysis on three RCTs consisting of placebo-controlled trials of six weeks duration (937 patients with paliperidone and 337 on placebo). In these studies, it was determined that 33% of the improvement in negative symptoms was due to the direct action of paliperidone, and the remainder was due to improvement in positive symptoms (51%), anxiety and depressive symptoms (18%) and movement disorders (2%). The study concluded that paliperidone produced effective action on negative schizophrenia symptoms.

Canuso et al.^33^ published an RCT comparing paliperidone with quetiapine and placebo among 399 patients hospitalized due to relapse of schizophrenia. Over the first two weeks of the study, the patients remained on monotherapy, and over the subsequent four weeks, the psychiatrists could combine another psychotropic drug, including an antipsychotic. At the end of the six weeks of the study, the patients in the paliperidone group had a significantly greater improvement in measurements such as PANSS (P = 0.023) than did patients using quetiapine, considering that the pattern of associations with other drugs was similar in both groups. Throughout the study, paliperidone was superior to quetiapine and placebo in improving negative symptoms (PANSS subscale, P < 0.005).

## QUETIAPINE IN TREATMENT-REFRACTORY SCHIZOPHRENIA

### Cochrane review on quetiapine for treating schizophrenia

Twelve RCTs were included in the review by Srisurapanont et al.,^12^ comprising a total of 3,443 individuals with schizophrenia.

**Figure 9.**
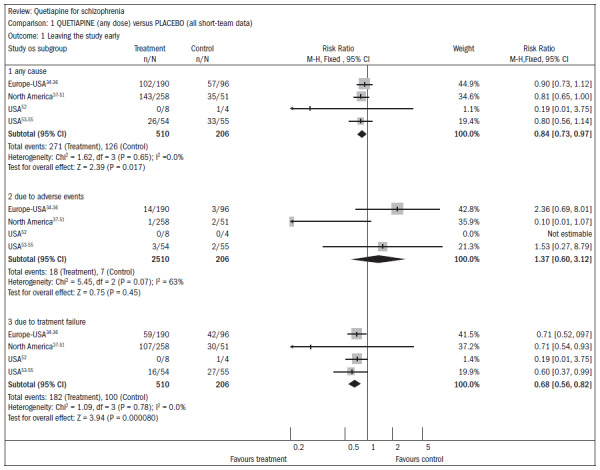
Quetiapine versus placebo, in relation to dropout rate in the study.

In four comparative studies with placebo (n = 716), there was a high dropout rate during the study: 53% loss with quetiapine and 61% for the placebo (RR 0.84; CI 0.7 to 0.9; NNT 11; CI 7 to 55), which limited the interpretation of other outcomes (Figure 9).^34-55^

**Figure 10.**
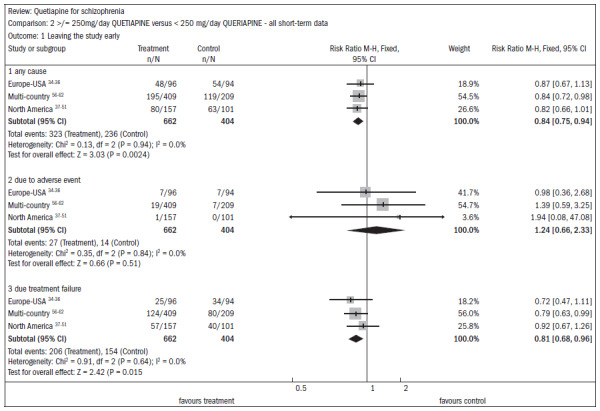
Higher doses of quetiapine (≥ 250 mg/day) versus lower doses of quetiapine (< 250 mg/day), in relation to dropout rate in the study.

In three studies (n = 1066) that compared higher doses of quetiapine (≥ 250 mg/day) with lower doses (< 250 mg/day), there were also high dropout rates in both groups. The group with higher doses had a 49% dropout rate, compared with 58% for lower doses (RR 0.84; CI 0.8 to 0.9; NNT 11; CI 7 to 29). It should be noted that there were two deaths in the group receiving the higher dose in one RCT (n = 618; RR 0.1; CI 0.0 to 2.1) (Figure 10).^34-51,56-62^

**Figure 11.**
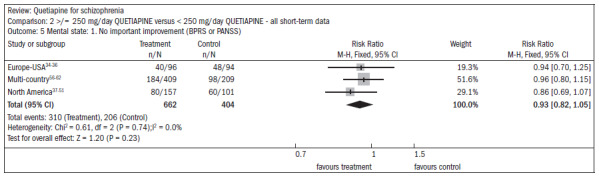
Higher doses of quetiapine (≥ 250 mg/day) versus lower doses of quetiapine (< 250 mg/day), in relation to mental state and lack of significant improvement.

There was a non-significant difference favoring the group receiving the higher doses of quetiapine, with regard to improving mental status (n = 1066; three RCTs; RR 0.93; CI 0.82 to 1.05) (Figure 11).^34-51,56-62^

**Figure 12.**
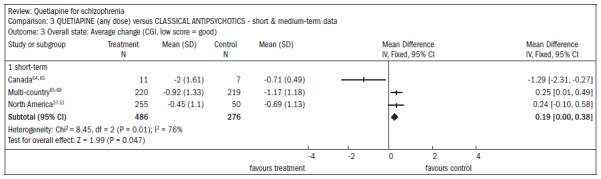
Efficacy of quetiapine versus classic antipsychotics, in relation to overall state, over the short and medium terms.

**Figure 13.**
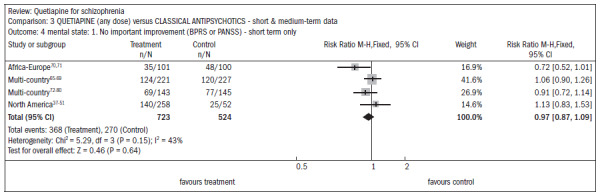
Efficacy of quetiapine versus classic antipsychotics, in relation to mental state and lack of significant improvement, over the short and medium terms.

**Figure 14.**
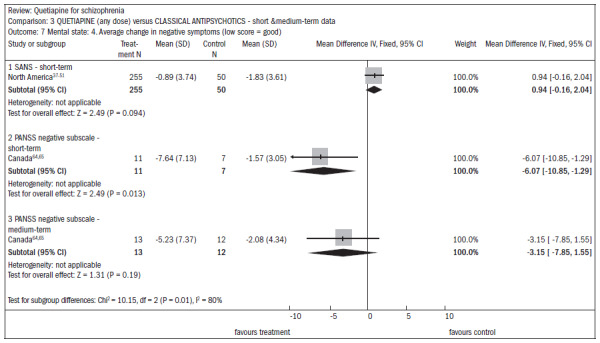
Efficacy of quetiapine versus classic antipsychotics, in relation to mental state and average change in negative symptoms.

In six studies comparing quetiapine with typical antipsychotics (n = 1924), the short-term dropout rate was around 36% in both groups (RR 0.87; CI 0.8 to 1.0). The outcomes related to symptomatic improvement in general, with no significant differences between quetiapine and typical antipsychotics:

- In assessing the overall improvement over the short and midterm, the results favored typical antipsychotics, despite the borderline statistical result (n = 762; DMP 0.2; CI 0.0 to 0.4) (Figure 12).^37-51,63-69^
- There was no difference in the improvement in mental status (n = 1247; RR 0.97; CI 0.9 to 1.1) (Figure 13).^37-51,65-80^
- There was no difference in the improvement in negative symptoms (n = 305; one RCT; DMP 0.94; CI -0.2 to 2.0) (Figure 14).^37-51,63,64^

**Figure 15.**
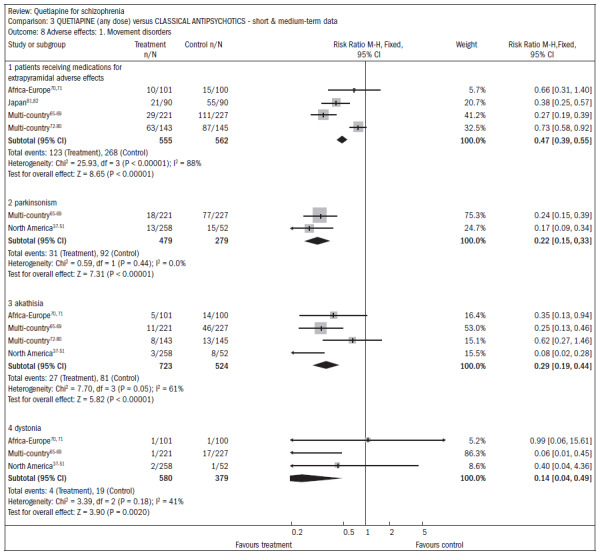
Quetiapine versus classic antipsychotics, in relation to movement disorders (adverse effects), over the short and medium terms.

Movement disorders were less prevalent among patients taking quetiapine, thus demonstrating the need for anti-Parkinson medication (n = 1117; four RCTs; RR 0.47; CI 0.4 to 0.6; NNT 4; CI 4 to 5; I^2^ 88%) (Figure 15).^37-51,65-82^

**Figure 16.**
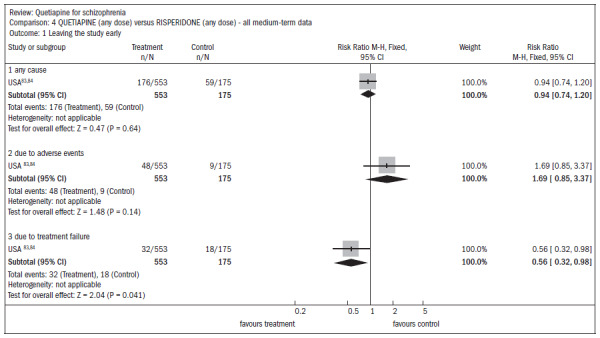
Quetiapine versus risperidone, in relation to dropout rate in the study.

Dry mouth (n = 649; two RCTs; RR 2.85; CI 1.5 to 5.6; NNH 17; CI 7 to 65) and somnolence (n = 959; three RCTs; RR 1.51; CI 1.1 to 2.2; NNH 18; CI 8 to 181) were more prevalent in the quetiapine group, compared with typical antipsychotics. One RCT compared quetiapine with risperidone, including 724 individuals with various psychotic symptoms, and two-thirds had a diagnosis of schizophrenia or schizoaffective disorder. Around 30% of the patients dropped out of the study before its end (n = 728; one RCT; RR 0.94; CI 0.7 to 1.2). Four people, all treated with quetiapine, died during the study (n = 728; one RCT; RR 2.86; CI 0.2 to 52.8) (Figure 16).^83,84^

**Figure 17.**
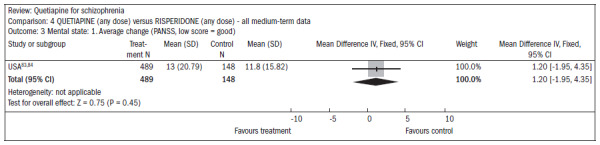
Quetiapine versus risperidone, in relation to mental state and average change.

All the evaluations of mental state outcomes were continuous and showed no statistical differences between quetiapine and risperidone, including the following (Figure 17):^83,84^

- reduced average PANSS score (n = 637; one RCT; MD, mean difference, 1.2; CI -2.0 to 4.4);
- reduction in PANSS subscale of positive symptoms (n = 639; one RCT; MD 0.70; CI -0.2 to 1.6);
- reduction in PANSS subscale of negative symptoms (n = 641; one RCT; MD 0.30; CI -0.8 to 1.4).

**Figure 18.**
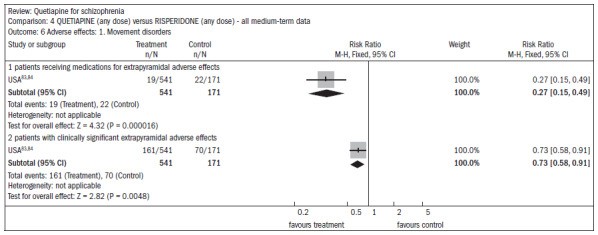
Quetiapine versus risperidone, in relation to movement disorders (adverse effects).

Extrapyramidal adverse events occurred less frequently in subjects receiving quetiapine than in subjects receiving risperidone, thus demonstrating the need for anti-Parkinson medication (n = 712; one RCT; RR 0.27; CI 0.2 to 0.5; NNT 11; CI 10 to 16), and in terms of the incidence of events (n = 712; one RCT; RR 0.73; CI 0.6 to 0.9; NNT 10; CI 6 to 28) (Figure 18).^83,84^

The following adverse events were more common with quetiapine than with risperidone:

- dizziness (n = 728; one RCT; RR 1.85; CI 1.0 to 3.3; NNH 18; CI 7 to 487);
- dry mouth (n = 728; one RCT; RR 2.11; CI 1.2 to 3.8; NNH 14; CI 6 to 82);
- drowsiness (n = 728; one RCT; RR 2.03; CI 1.4 to 2.9; NNH 7; CI 4 to 17).

### Conclusions from review

Quetiapine is effective for treating schizophrenia, but not very different from typical antipsychotics or risperidone in terms of efficacy and relapse prevention, but it has a lower risk of movement disorders and higher risk of dizziness, dry mouth and drowsiness. There was a recommendation for further studies to evaluate the effectiveness of this drug.

### Studies on refractory schizophrenia

Despite an extensive search of the literature, only two RCTs including patients with refractory episodes were found to have evaluated quetiapine. In the study by Conley et al.,^85^ a comparison was made between risperidone (4 mg/day), quetiapine (400 mg/day) and fluphenazine (12.5 mg/day) over a 12-week period on a sample of 38 individuals. The dropout rate from treatment was greater with fluphenazine (69%) than with risperidone (31%) and quetiapine (42%). The criterion of response to treatment (reduction in BPRS scale > 20%) was achieved by 23% of those taking risperidone, 25% with quetiapine and 15% with fluphenazine.

Genç et al.^86^ reported on an RCT that evaluated 56 patients who were resistant to treatment and partially responsive to clozapine. The combination of clozapine plus amisulpride was compared with clozapine plus quetiapine. At the end of eight weeks of study, both groups improved significantly, but the improvement was greater with amisulpride than with quetiapine. This difference in favor of amisulpride was shown from the third week onwards in assessments on the CGI scale, and from the sixth week onwards using the BPRS, SANS and SAPS rating scales. The two association strategies were well tolerated.

## RISPERIDONE FOR TREATING REFRACTORY SCHIZOPHRENIA

Risperidone is a benzisoxazole derivative, with a strong blocking effect on D2 and 5-HT2 receptors. It binds to a1, a2 and H1 receptors, and is also a potent LSD (lysergic acid diethylamide) antagonist. It is, however, virtually devoid of anticholinergic effects.

### Cochrane review of risperidone versus typical antipsychotics for treating schizophrenia

**Figure 19.**
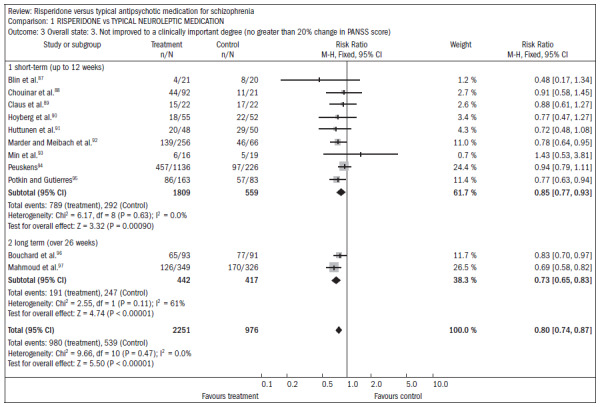
Risperidone versus typical neuroleptic medication, in relation to overall state and lack of clinically significant degree of improvement.

The review by Hunter et al.^13^ included 23 RCTs with 4,445 individuals, and evaluated the effectiveness of risperidone versus typical antipsychotics for treating schizophrenia. Over the short term, risperidone was more effective in reducing positive and negative symptoms, according to PANSS, compared with haloperidol (n = 2368; nine RCTs; RR 0.72; CI, 20% did not improve, 0.59 to 0.88; NNT 8) (Figure 19).^87-97^

The favorable outcome for risperidone was also sustained in long-term studies (n = 859, 2RCTs RR, 20% did not improve, 0.51 CI; 0.38 to 0.67; NNT 4; n = 675; one RCT; RR, not improved 40%, 0.75; CI 0.66 to 0.84; NNT 5; n = 675, one RCT; RR, 60% not improved, 0.90; CI 0.84 to 0.96; NNT 11). A follow-up on the patients performed one year after administering risperidone also found a lower recurrence rate, compared with haloperidol use (n = 367; one RCT; RR 0.64; CI 0.41 to 0.99; NNT 7). Patients taking risperidone showed significantly fewer movement disorders overall (including extrapyramidal side effects) than did those receiving older typical antipsychotics (n = 2702; 10 RCTs; RR 0.63; CI 0.56 to 0.71; NNT 3). The number of patients taking antiparkinsonian drugs was significantly lower in the risperidone group (n = 2524; 11 RCTs; RR 0.66; CI 0.58 to 0.74; NNT 4). In relation to body weight, four studies (n = 1708) found a higher propensity for weight gain among patients using risperidone, compared with those taking typical antipsychotics (RR 1.55; CI 1.25 to 1.93; NNH 3).

There was no difference between the two drugs regarding the number of patients with erectile dysfunction (n = 106; two RCTs; RR 1.55; CI 0.58 to 4.20). Some studies have shown that patients who took risperidone reported rhinitis more frequently than did those taking conventional antipsychotics (n = 656; three RCTs; RR 1.99; CI 1.24 to 3.19; NNH 3).

### Conclusions from review

Risperidone appears to be more acceptable for patients with schizophrenia than are typical antipsychotics. The side effects are less frequent compared with haloperidol. The benefits achieved with risperidone should be analyzed taking its higher cost into consideration, as well as its tendency to promote weight gain. Recent data regarding the reduction of relapse rates with risperidone over the long term, compared with typical antipsychotics, need to be replicated in other studies.

### Cochrane review of risperidone versus atypical antipsychotics for treating schizophrenia

The systematic review by Gilbody et al.^14^ included nine studies comparing risperidone with clozapine (five studies involving patients with criteria for refractory schizophrenia), olanzapine (three studies) and amisulpride (one study). The dropout rates were high. The total PANSS scores were not significantly different (n = 134; DMP 2.0; CI -3.04-6.97) in the assessment six weeks after starting the treatment, and studies that had sufficient data to allow evaluations on segregated positive and negative subscales showed no significant difference (PANSS positive scale, n = 134; DMP 1.359; CI -0.581 to 3.320; PANSS and BPRS negative symptoms subscale, n = 134; DMP -0.17; CI -0.521 to 0.163). The wide confidence intervals did not allow assignation of equivalence between risperidone and clozapine, or determination of superiority of either of these drugs. Overall scores, as measured on the CGI scale, showed no differences between the groups (n = 105; DMP 0.00; CI -0.46 to 0.47). There was no difference over the short term in relation to tolerance to treatment, reported by patients using clozapine and risperidone (n = 466; RR 1.00; CI 0.73 to 1.37).

Olanzapine and risperidone appear to be very similar with regard to the number of patients who responded to treatment (40% reduction in PANSS, n = 339; RR 1.14; CI 0.99 to 1.32). Patients taking olanzapine adhered better to the study protocol (n = 404; RR 1.31; CI 1.06 to 1.60; NNT 8; CI 4 to 32) and had fewer side effects and extrapyramidal effects (n = 339; RR 1. 67; CI 1.14 to 2.46; NNH 8; CI 5 to 33) although the doses of risperidone administered were higher than those recommended in practice. The doses administered in these studies ranged from 1 mg/day to 16 mg/day. In one study (n = 228), amisulpride seemed to be very similar to risperidone in most parameters. No useful data was presented regarding the cost of treatment. The data presented on quality of life was also very scarce.

### Conclusions from review

Equivalence between clozapine and risperidone for treating refractory schizophrenia may not yet have been established. There seem to be few differences between olanzapine, risperidone and amisulpride. The studies available are limited in many ways: lacking long-term follow-up, clinically relevant parameters, and assessment of quality of life. For individuals with mild schizophrenia, risperidone is an effective antipsychotic, but can cause movement disorders and more side effects than other new drugs such as olanzapine, although this figure was based on a trial from a study using higher doses of risperidone than those used in clinical practice. Olanzapine, risperidone and clozapine tend to lead to weight gain, while risperidone may be preferable. More research is needed to confirm these findings. In cases of refractory schizophrenia, clozapine is often offered as an alternative but it has significant side effects that require weekly laboratory monitoring. Risperidone does not appear to be as acceptable as clozapine regarding occurrences of movement disorders, and this is the only reason clozapine is usually offered after risperidone. However, there is still insufficient evidence to suggest that risperidone is as effective as clozapine for symptoms refractory to typical antipsychotics.

### Cochrane review of risperidone compared with olanzapine for treating schizophrenia

**Figure 20.**
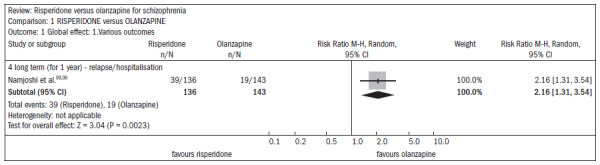
Efficacy of risperidone versus olanzapine, in relation to relapse/hospitalization.

**Figure 21.**
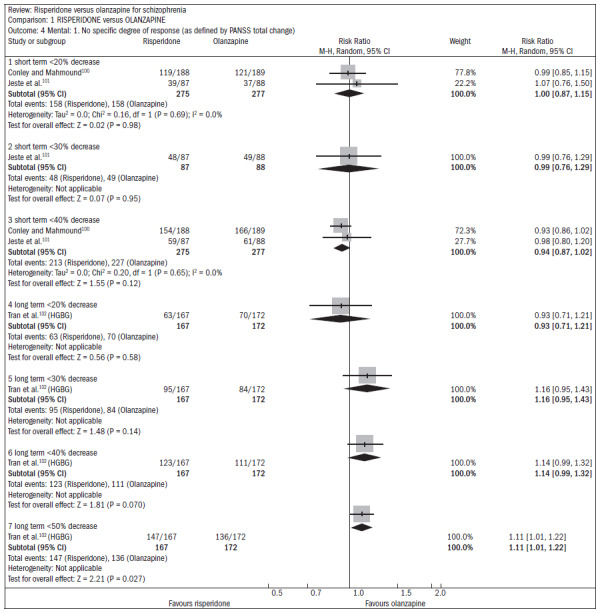
Efficacy of risperidone versus olanzapine, in relation to mental state/no specific degree of response.

Over the short term, no difference was found between risperidone and olanzapine with regard to symptom improvement (n = 548; two RCTs; RR 1.00; CI 0.88 to 1.15).^15^ One study showed favorable results with olanzapine regarding the outcome of relapse/rehospitalization over a 12-month period, compared with risperidone (n = 279; one RCT; RR 2.16; CI 1.31 to 3.54; NNH 7; CI 3 to 25) (Figure 20).^98,99^ There was no difference between risperidone and olanzapine regarding mental state parameters evaluated using the NSSHL scale (n = 552; two RCTs; RR "no PANSS decrease of 20% for eight weeks", 1.01; CI 0.87 to 1.16) (Figure 21).^100-102^

**Figure 22.**
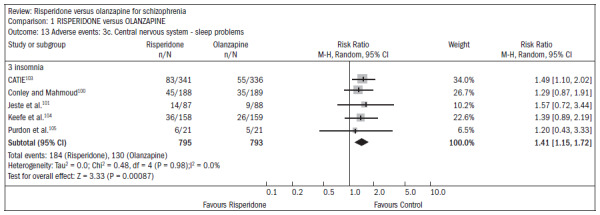
Risperidone versus olanzapine, in relation to sleep problems/insomnia.

**Figure 23.**
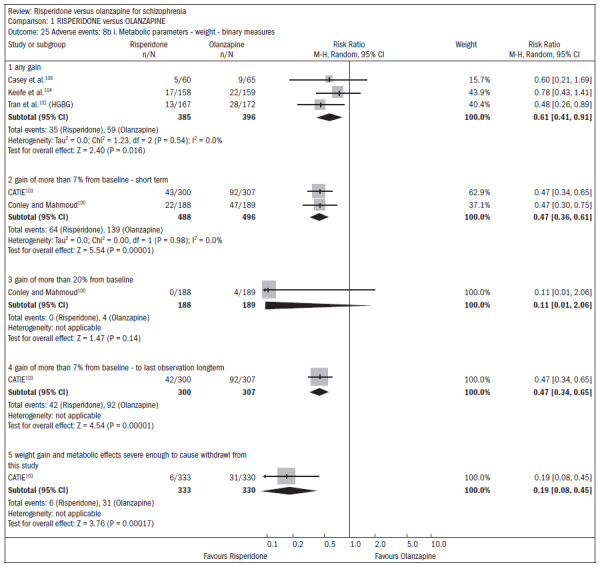
Risperidone versus olanzapine, in relation to metabolic parameters/weight.

Both drugs caused adverse events: 75% of the patients reported some event, and 20% reported anticholinergic symptoms. Both groups reported insomnia, although it was more frequent among patients taking risperidone (n = 1588; five RCTs; RR 1.41; CI 1.15 to 1.72; NNH 15; CI 9 to 41) (Figure 22).^100,101,103-105^ About 30% of the patients reported somnolence (n = 1713; six RCTs; RR 0.92; CI 0.79 to 1.07). With regard to extrapyramidal symptoms (n = 893; three RCTs; RR 1.18; CI 0.75 to 1.88), 25% of the subjects taking risperidone required the use of additional medication to relieve these symptoms (n = 419; two RCTs; RR 1.76; CI 1.25 to 2.48; NNH 8; CI 4 to 25). Patients taking risperidone were less prone to weight gain, compared with those taking olanzapine. Weight gain was significant (n = 984; two RCTs; RR "gain of more than 7% of baseline weight over the short term", 0.47; CI 0.36 to 0.61; NNH 7; CI 6 to 10) Patients taking risperidone were less likely to abandon treatment because of metabolic side effects and weight gain, compared with those taking olanzapine (n = 667; one RCT; RR 0.19; CI 0.08 to 0.45) (Figure 23).^100,102-104,106^

Patients taking risperidone more frequently presented ejaculation disorders (n = 370; two RCTs; RR 4.36; CI 1.38 to 13.76; NNH 20; CI 6 to 176). Both drugs were associated with high dropout rates over time: 66% in the risperidone group compared with 56% in the olanzapine group (n = 1440; five RCTs; RR 1.17; CI 1.08 to 1.27; NNH 11; CI 7 to 23).

### Conclusions from review

Both drugs are associated with reducing the levels of psychotic symptoms. One study showed favorable results with olanzapine regarding the outcome of relapse/rehospitalization over a 12-month period, compared with risperidone. Occurrences of side effects were frequent with both drugs. Some differences were found in relation to the types of side effects. Risperidone and olanzapine are expensive drugs. Patients should be informed about the side effects.

### Cochrane review of long-acting injection of risperidone for treating schizophrenia

**Figure 24.**
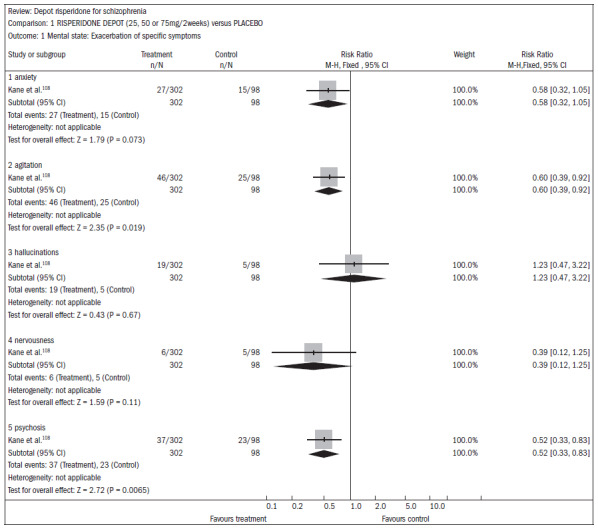
Injectable risperidone versus placebo, in relation to mental state/exacerbation of specific symptoms.

A systematic review published in the Cochrane Library^107^ evaluated the effectiveness of long-acting risperidone injections for treating schizophrenia. Only one study (n = 400) was found comparing risperidone with a placebo injection, but 56% of the patients did not complete the three-month study, thus rendering some of the data on the overall effect and mental state unusable. Injectable risperidone (25 mg, 50 mg and 75 mg) compared with placebo did not affect anxiety levels (n = 400; RR 0.58; CI 0.32 to 1.05) but decreased agitation (n = 400; RR 0.60; CI 0.39 to 0.92). Injectable risperidone did not significantly affect hallucination episodes (n = 400; RR 1.23; CI 0.47 to 3.22), but the number of episodes of psychosis was diminished (n = 400; RR 0.52; CI 0.33 to 0.83; NNT 9; CI 7 to 26) (Figure 24).^108^

**Figure 25.**
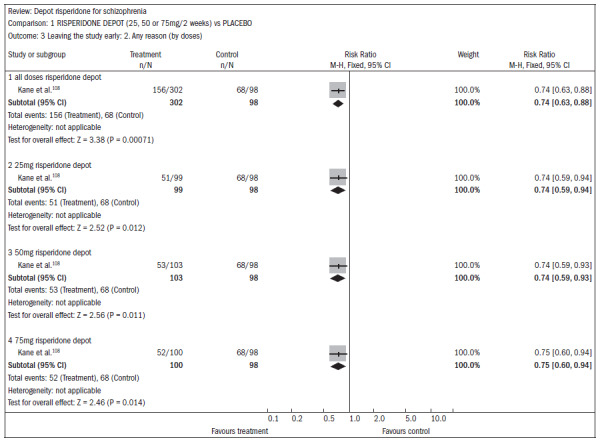
Injectable risperidone versus placebo, in relation to dropout from the study reported for any reason.

The dropout rates were higher in the treatment group using injectable risperidone than in the placebo group (n = 400; RR 0.74; CI 0.63 to 0.88; NNT 6; CI 4 to 12) (Figure 25).^108^

Severe side effects were common in both groups (13% to 23%), but more significant in the placebo group (n = 400; RR 0.59; CI 0.38 to 0.93; NNT 11; CI 7 to 70). However, incomplete records made the data difficult to interpret. Movement disorders were common in both groups (n = 400; RR 2.38; CI 0.73 to 7.78), although it seemed that they were more frequent among patients taking injectable risperidone at higher doses. Only one study (n = 640) compared injectable risperidone to oral risperidone among patients with mild schizophrenia. For the overall state, there were no differences between the two approaches (n = 640; RR "no overall improvement" 1.06; CI 0.92 to 1.22). Mental state measurements were also similar between the groups. Just over half of both groups reported adverse events (n = 640; RR 1.04; CI 0.91 to 1.18).

### Conclusions from review

This review identified only two studies. It was concluded that among less stable patients, the need for regular oral doses could be avoided through the use of injectable risperidone, although the events are still not well reported. In severe cases of schizophrenia, some benefits are obvious, with increased rates of adherence to treatment with injectable risperidone, compared with placebo.

### Studies on refractory schizophrenia

Four RCTs assessing risperidone for treating refractory schizophrenia were included in the new search. Suzuki et al.^109^ evaluated the effectiveness of an antipsychotic polypharmacy regimen for treating refractory schizophrenia. Seventeen patients with refractory schizophrenia who did not respond to a monotherapy sequence of olanzapine, quetiapine and risperidone were subsequently treated with combination therapy consisting of olanzapine plus risperidone for at least eight weeks. Seven patients responded to the combination treatment in accordance with the primary endpoint, which was defined as post-treatment measurements on the BPRS of less than 70% of the pretreatment values. These patients were classified as such, on average 10 weeks after starting polypharmacy. Two were successful in subsequent conversion to monotherapy. None of the patients stopped treatment prematurely. The overall functioning assessment score improved from 37.1 to 53.0 among the patients who responded to treatment (mean maximum dose: 12.9 mg of olanzapine and 3.14 mg of risperidone), while it did not change significantly among the other patients (mean maximum dose: 14.5 mg of olanzapine and 5.50 mg of risperidone). Body weight, prolactin and total cholesterol increased significantly.

The present authors concluded that an antipsychotic polypharmacy scheme might be useful for refractory patients, but the increase in adverse events would need to be considered. New studies to evaluate combination therapy consisting of antipsychotics versus clozapine or tenacious long-term monotherapy strategies would be needed to assess the response to refractory schizophrenia.

Freudenreich et al.^110^ conducted a randomized parallel clinical double-blind trial evaluating the effectiveness of risperidone in combination with clozapine for treating refractory schizophrenia. Twenty-four patients received a fixed dose of risperidone 4 mg/day for six weeks. The patients who received risperidone did not show any significant decrease in total PANSS scores. The PANSS subscale evaluation improved significantly (P = 0.047). The authors concluded that, according to the results obtained from this study, there were no additional benefits from the association of risperidone and clozapine among patients with refractory schizophrenia. However, further trials would be needed in order make a definitive assessment regarding the efficacy of this combination.

Lewis et al.^111^ conducted a randomized multicenter study in the United Kingdom in partnership with the National Health Service (NHS). The study included 136 participants aged 18-65 years diagnosed with schizophrenia according to the DSM-IV whose medication was changed because of lack of clinical response from the use of two or more antipsychotics. The participants were randomly assigned risperidone, clozapine, olanzapine, quetiapine or amisulpride. The results were evaluated by assessors who were blinded to the allocation group. A one-year follow-up was conducted on 87% of the sample. Intention-to-treat analysis showed that there was no statistically significant difference in the scores on the quality-of-life scale regarding treatment with clozapine (3.63 points; CI: 0.46 to 7.71; P = 0.08), but that there was a statistically significant advantage according to the total PANSS score (-4.93 points; CI -8.82 to -1.05; P = 0.013) at the one-year follow-up. Users of clozapine reported a lower rate of extrapyramidal side effects. In the 12^th^ week, the participants who received clozapine reported that their mental health was significantly better, compared with participants receiving other antipsychotics. The authors concluded that for patients with schizophrenia who did not respond to two or more antipsychotic medications, there was an advantage in treating with clozapine, in terms of improvement of symptoms over a one-year period.

Honer et al.^112^ conducted a randomized clinical double-blind trial to evaluate patients with schizophrenia who did not respond to treatment with clozapine. Patients continued to take clozapine and were randomly assigned to receive 3 mg of risperidone daily for eight weeks or a placebo. This course of treatment was followed by an optional additional 18 weeks with risperidone. The main outcome evaluated was the reduction in the total score attributed to the severity of positive and negative symptoms, as measured by PANSS. Secondary outcomes included cognitive functioning. Sixty-eight patients were randomly assigned to treatment. In the double-blind phase, the mean total score for the severity of symptoms decreased over the eight-week evaluation for both groups (risperidone and placebo). There was no statistically significant difference regarding the symptomatic benefit from associations with risperidone and placebo: nine out of the 34 patients who received placebo and six out of the 34 who received risperidone responded to treatment (P = 0.38). The mean difference in PANSS score change from baseline to the eighth week, between those receiving risperidone and those receiving placebo was 0.1 (95% CI -7.3 to 7.0). The verbal working memory index showed a slight decline in the risperidone group and a small improvement in the placebo group (P = 0.02), comparing the changes in the two groups from baseline. The increase in blood glucose levels was higher in the risperidone group than in the placebo group. There was no difference between the groups regarding occurrence and severity of other side effects. The authors of the study concluded that there were no additional benefits from associating risperidone and clozapine among patients with severe schizophrenia.

## DISCUSSION

This systematic review presents a broad overview of randomized controlled trials and systematic Cochrane reviews that have been conducted in relation to the use of aripiprazole, paliperidone, quetiapine and risperidone for treating schizophrenia. There have been few studies in the literature on treatments for refractory patients. Only a single Cochrane review examined nine studies comparing risperidone with clozapine for treating refractory patients.^14^ The present paper has now updated that review, with the addition of 10 RCTs as follows: two RCTs (aripiprazole), two RCTs (paliperidone), two RCTs (quetiapine) and four RCTs (risperidone). Among the drugs evaluated, risperidone has been on the market longer than other antipsychotics and has the best data for demonstrating its effectiveness in this subgroup of patients.

In comparing these four atypical antipsychotics with first-generation or typical antipsychotics, the main advantage of atypical antipsychotics, which is common to all drugs in this category, is their favorable safety profile with regard to adverse extrapyramidal events. Side effects such as akathisia, parkinsonism and tardive dyskinesia often limit the long-term use of typical antipsychotics, thus leading to abandonment of treatment and a high relapse rate. On the other hand, it is important to emphasize that the clinical trials included in this review, which were mostly short and medium-term studies, have not demonstrated clear advantages in treatment compliance.

Regarding comparisons between atypical antipsychotics, the data are even more limited. The main drugs compared in clinical trials, some of which are specific to refractory schizophrenia, included amisulpride, clozapine, risperidone and olanzapine, because these are the drugs that demonstrate improved efficacy profiles, compared with typical antipsychotics, thereby demonstrating advantages in terms of improving negative symptoms, for example.^113^ Likewise, users of risperidone and paliperidone have a greater propensity towards extrapyramidal events, which are potentially dose-dependent reactions.

A systematic review by Leucht et al.^113^ evaluated comparisons between atypical antipsychotics alone that were identified in 78 RCTs with 13,558 participants. Its main conclusions were that: risperidone is more effective than quetiapine; ziprasidone olanzapine is more effective than aripiprazole, quetiapine, risperidone and ziprasidone (separately); and clozapine, at doses above 400 mg/day, is more effective than risperidone. These authors also concluded that, despite some differences in negative symptoms, the benefits (from atypical antipsychotics) basically relate to efficacy in dealing with positive symptoms of schizophrenia. One important matter that has been extensively investigated in the literature on the role of antipsychotics is long-term compliance.

A study conducted in Brazil by Rosa et al.^114^ showed that after one year, 50% of patients discontinued the treatment. Over recent years, some studies in other countries, have taken the view that adherence to treatment should be the main endpoint for measuring the effectiveness of treatment, because this includes an assessment of the balance between the benefits (effectiveness) and risks to safety of these interventions.

The CATIE study (Clinical Antipsychotic Trials of Intervention Effectiveness),^103^ conducted by the NIMH (National Institute of Mental Health, United States), included 1,460 individuals with schizophrenia in 57 centers in the United States, and patients with a first episode or refractory episodes were excluded. The percentages of patients who had discontinued treatment by the 18^th^ month were: 64% on olanzapine (mean dose 20.1 mg/day, n = 336); 74% on perphenazine (20.8 mg, n = 245); 75% on quetiapine (543.4 mg, n = 309); 79% on risperidone (3.9 mg, n = 305); and 82% ziprasidone (112.8 mg, n = 165). Patients taking olanzapine adhered better to treatment than did those taking quetiapine (P < 0.001) and risperidone (P = 0.002).

The EUFEST study (European First Episode Schizophrenia Trial)^115^ was a pragmatic multicenter study in 13 European countries and Israel, which evaluated 498 patients with newly diagnosed schizophrenia (less than two years) and was sponsored by AstraZeneca, Pfizer and Sanofi-Aventis. The rate of discontinuation of treatment after one year was 22% with amisulpride (200-800 mg, n = 104); 34% with haloperidol (1-4 mg, n = 103); 22% with olanzapine (5-20 mg, n = 105); 33% with quetiapine (200-750 mg, n = 104); and 36% with ziprasidone (40-160 mg, n = 82). Dropouts among patients on haloperidol occurred earlier than with other drugs (P < 0.01).

In addition to the choice of depot antipsychotic medications, quarterly or monthly use can also be a significant factor in improving adherence to treatment among individuals with schizophrenia. Antipsychotics with prolonged action, such as haloperidol decanoate, pipotiazine, zuclopenthixol and penfluridol, have been important therapeutic options since the 1960s. They act by keeping the plasma levels of the active agent stable, thereby improving adherence and preventing relapses. In fact, relapse prevention is one of the main targets in treating schizophrenia and is therefore considered to be one of the most important outcome criteria used to assess the effectiveness of an antipsychotic drug. However, it is well established that chronic use of first-generation antipsychotics may be associated with the development of extrapyramidal effects, (e.g. tardive dyskinesia), and in this sense, conventional depot antipsychotics may not be the best treatment option, despite their proven efficacy. On the other hand, it has already been shown that long-term use of second-generation antipsychotics is associated with a lower risk of developing extrapyramidal symptoms. Thus, use of second-generation depot antipsychotics, such as risperidone, for patients who adhere to treatment, represents a substantial improvement in combating the development of schizophrenia.

Combinations of antipsychotics for refractory cases of schizophrenia have also been used, and studies have shown some clinical benefits, but also a deterioration of tolerability. A systematic review published in 2009^116^ evaluated association strategies, and demonstrated that the benefits occurred mainly in relation to associations between typical and atypical antipsychotics, and not in relation to associations between two typical antipsychotics or between two atypical antipsychotics. These data have not been confirmed, but may be relevant to clinical practice (Table 1).^116^

**Table 1.** Efficacy of associations between antipsychotics^1116^

Antipsychotics	Patients	RR(95% CI)	NNT (95% CI)	P value
Typical versus atypical	438	ns	–	0.37
Atypical + atypical versus typical	336	ns	–	0.65
Atypical + typical versus typical	171	0.47 (0.22-0.98)	3 (2-9)	0.004
Atypical + typical versus atypical	257	0.59 (0.40-0.88)	3 (2-6)	< 0.0001

RR = relative risk of antipsychotics; 95% CI = 95% confidence interval; NNT = number needed to treat; ns = non-significant.

Finally, clozapine remains the only drug specifically indicated for treating refractory schizophrenia according to the British NHS. Therefore, clinicians should consider this to be an option for patients presenting resistance to treatment. Certain serious medical conditions relating to schizophrenia are particularly sensitive to clozapine, including persistent auditory hallucinations, persistent hostility, suicide risk and tardive dyskinesia. The main limitation on the use of clozapine is the need for monitoring and prevention of agranulocytosis, which affects 1% of patients for which patients need to have blood samples drawn regularly.

## CONCLUSIONS

In treating schizophrenia, the antipsychotics quetiapine, aripiprazole and paliperidone were not associated with statistically significant differences in outcome improvement regarding overall psychopathology, as measured using scales such as PANSS, BPRS or CGI, in comparison with first-generation antipsychotics. Risperidone was specifically evaluated among patients with refractory schizophrenia in one of the systematic reviews included here, and this demonstrated favorable but not definitive outcomes, compared with drugs that have also been shown to be effective, such as amisulpride, clozapine and olanzapine. A lower frequency of extrapyramidal side effects has been reported in RCTs that evaluated quetiapine, aripiprazole, paliperidone and risperidone in comparison with first-generation antipsychotics.

### Implications for clinical practice

Although there are several antipsychotics on the market and new drugs are released regularly, the population of patients with refractory schizophrenia still remains high. These people present limited response to antipsychotics and persist with residual psychotic symptoms. Treatment with first and second-generation antipsychotics may have similar efficacy; however, patients treated with atypical antipsychotics are more likely to adhere to treatment. Investigation of dose adequacy and even the particular combinations of antipsychotics are also among the actions that can provide benefits for some patients.

### Implications for research

There remains a need for studies to evaluate the effectiveness of interventions for patients with refractory schizophrenia. Outcomes of risk profile, long-term benefit, relapse rates, residual symptoms, safety and cost-effectiveness of antipsychotics need to be better established. Among the outcomes to be studied, treatment compliance is an indicator that assesses the balance between efficacy and safety. It is a good means of assessing the overall effectiveness of an antipsychotic drug and should be considered a primary outcome. Quality of life, wellbeing and all assessments of personal and family interventions also need to be better evaluated. Studies on depot antipsychotics, and particularly atypical antipsychotics, are very relevant for such patients. Finally, studies on associations between antipsychotics are very relevant in this situation of such limited information on clinical outcomes.
